# Heat Stroke Encephalopathy Without the Presence of Distinct Imaging Findings: Case Report and Review of Literature

**DOI:** 10.1155/crcc/6609879

**Published:** 2026-06-07

**Authors:** Ryan Sholes, David J. Walz, Julia Evans, Tessa Connelly, Joseph Rigotti

**Affiliations:** ^1^ Sidney Kimmel Medical College, Philadelphia, Pennsylvania, USA, jefferson.edu; ^2^ Jefferson Abington Hospital, Abington, Pennsylvania, USA, jeffersonhealth.org

## Abstract

Heat stroke is a life‐threatening emergency characterized by severe hyperthermia and acute central nervous system (CNS) dysfunction. We describe a 72‐year‐old man who was found unresponsive in his vehicle on a day with ambient temperatures exceeding 90°F. On arrival, his core temperature was 105.8°F, and Glasgow Coma Scale (GCS) score was 5. Despite prompt initiation of active cooling and supportive care with normalization of body temperature, the patient developed persistent and fluctuating encephalopathy. Extensive metabolic, infectious, and toxicologic evaluations were unrevealing. Thyroid‐stimulating hormone was normal, arterial blood gases showed no acid–base derangements, ammonia and liver function tests were within normal limits, and urine toxicology was negative. Electroencephalography demonstrated diffuse cerebral slowing without epileptiform activity. Brain MRI performed approximately 1 week after admission under anesthesia, including diffusion‐weighted imaging, showed no acute abnormalities, revealing only mild age‐related atrophy and chronic small‐vessel ischemic changes. The patient′s neurologic function failed to recover over a prolonged hospitalization, and he ultimately died following transition to comfort‐focused care on hospital Day 27. This case highlights that severe and persistent encephalopathy may occur in the setting of probable heat stroke despite unrevealing conventional neuroimaging and underscores the diagnostic uncertainty and limitations of MRI in evaluating heat‐related neurologic injury.

## 1. Introduction

Heat stroke is a life‐threatening condition defined by hyperthermia, typically exceeding 40°C (104°F), accompanied by acute CNS dysfunction [1, 2]. It is broadly categorized as exertional or nonexertional, with the latter more commonly affecting older adults and individuals with chronic medical conditions [3]. Neurologic manifestations range from delirium and seizures to coma, and long‐term cognitive and motor deficits have been reported [2, 4].

Persistent or fluctuating encephalopathy following heat stroke has been described in a limited number of case reports, most of which document characteristic abnormalities on brain MRI, particularly diffusion‐weighted imaging [5–12]. These findings commonly include cerebellar involvement, hippocampal lesions, or diffuse cortical injury. However, the absence of acute neuroimaging abnormalities does not preclude significant neurologic dysfunction. We report a case of probable nonexertional heat stroke with prolonged encephalopathy and unrevealing MRI findings, highlighting the diagnostic challenges and limitations of neuroimaging in this context.

## 2. Case Presentation

A 72‐year‐old man with a history of depression, anxiety, and mild cognitive impairment was brought to the emergency department after being found unresponsive in his parked vehicle. Ambient temperatures on the day of presentation ranged from 31°C to 33°C (88°F–92°F). Emergency medical services initiated external cooling prior to arrival. On presentation, his core temperature was 41°C (105.8°F), heart rate 104 beats per minute, blood pressure 137/66 mmHg, and oxygen saturation 98% on 2‐L nasal cannula. His GCS score was E1/V1/M3, and he was minimally responsive to painful stimuli.

Active cooling was continued with ice packs, ice‐water lavage, and Foley catheter placement for temperature monitoring. Rhythmic, nonpurposeful jerking movements were observed, and 2 mg of intravenous lorazepam was administered. His mental status transiently improved to GCS E2/V2/M3 with confabulatory speech. According to family, the patient lived independently and was typically alert and oriented at baseline.

Initial laboratory evaluation demonstrated sodium 137 mmol/L, potassium 3.7 mmol/L, bicarbonate 20 mmol/L, creatinine 0.51 mg/dL, creatine kinase 42 IU/L, white blood cell count 5.7 B/L, hemoglobin 13 g/dL, and lactate 1.5 mmol/L. Thyroid‐stimulating hormone was normal at 1.15 *μ*IU/mL. Liver function tests and ammonia level (28 *μ*mol/L) were within normal limits. Arterial and venous blood gases revealed no significant acid–base disturbance (ABG pH 7.44/PaCO₂ 41/PaO₂ 104/HCO₃^−^ 28; VBG pH 7.41/PCO₂ 32/PO₂ 54/HCO₃^−^ 22). Urine toxicology, salicylates, and ethanol levels were negative. Home medications included fluoxetine 20 mg daily, with no other psychoactive or anticholinergic agents identified.

Computed tomography of the head showed mild chronic small‐vessel ischemic changes and age‐related volume loss without acute findings. Over the first several days, body temperature normalized; however, encephalopathy persisted. Sodium gradually increased late in the hospitalization, peaking at 151 mmol/L in the setting of poor oral intake. The patient developed transient leukocytosis later in his course (peak 19.5 B/L), which resolved prior to discontinuation of laboratory monitoring.

An extensive infectious evaluation was pursued. Blood cultures remained negative. Cerebrospinal fluid analysis, performed approximately 1 week after admission due to severe agitation requiring anesthesia, demonstrated normal glucose, mildly elevated protein, no pleocytosis, and negative bacterial, viral, and fungal studies. Broad‐spectrum antibiotics and acyclovir were initiated empirically; acyclovir was discontinued after creatinine rose to a peak of 2.4 mg/dL, subsequently improving to 0.64 mg/dL with cessation of therapy.

Neurology consultation was obtained. Routine EEG performed on hospital Day 5 demonstrated diffuse cerebral slowing without epileptiform discharges or seizures. Brain MRI was completed approximately 1 week after admission under anesthesia and included sagittal T1, axial T2, axial FLAIR, gradient echo, diffusion‐weighted imaging, and postcontrast sequences with intravenous gadolinium. No acute infarction, diffusion restriction, hemorrhage, mass lesion, or abnormal enhancement was identified. Findings were limited to mild generalized atrophy and chronic microvascular changes.

The patient′s mental status fluctuated throughout hospitalization, intermittently improving to GCS E4/V4/M6 before recurrent deterioration. Total lorazepam exposure was limited to 5 mg over 27 days; episodes of worsened confusion were temporally associated but not exclusively explained by sedative administration. Due to persistent inability to protect his airway and inadequate oral intake, a percutaneous endoscopic gastrostomy tube was placed, which the patient subsequently removed. After multidisciplinary discussions, the family elected not to pursue replacement and transitioned the patient to comfort‐focused care. He died on hospital Day 27 without a discrete terminal event.

## 3. Discussion

Heat stroke represents a transition from compensable thermoregulation to a noncompensable state in which heat production exceeds dissipation, resulting in systemic inflammation, endothelial dysfunction, and multiorgan injury [2, 13]. Neurologic involvement is a defining feature and may result from cytokine‐mediated blood–brain barrier disruption, hypoperfusion, oxidative stress, and direct thermal cytotoxicity, particularly affecting cerebellar Purkinje cells [6, 14, 15].

Persistent encephalopathy following heat stroke has been reported primarily in cases demonstrating characteristic MRI abnormalities, including diffusion restriction in the cerebellum, hippocampi, thalami, and cerebral cortex [5–12]. In contrast, our patient demonstrated severe and prolonged neurologic dysfunction despite unrevealing conventional MRI obtained approximately 1 week after presentation. While delayed imaging raises the possibility that transient diffusion abnormalities were missed, prior reports suggest that MRI changes may persist for weeks in many cases, indicating that the absence of findings cannot be solely attributed to timing.

Alternative etiologies of encephalopathy were considered. Thyroid function was normal, making myxedema coma unlikely. Liver dysfunction, hyperammonemia, and significant metabolic derangements were absent. Although infectious encephalitis could not be definitively excluded due to delayed lumbar puncture and prior antimicrobial therapy, extensive testing and lack of systemic inflammatory markers reduced this likelihood. Sepsis‐associated encephalopathy was also considered; however, the absence of sustained hemodynamic instability, culture positivity, or progressive organ failure argues against it as the primary cause.

Diffuse EEG slowing, while nonspecific, is consistent with severe global cerebral dysfunction and has been reported in heat stroke–associated encephalopathy. Continuous EEG monitoring was not performed, limiting exclusion of intermittent nonconvulsive seizure activity. Sedative exposure likely contributed to delirium and fluctuations in mental status but does not fully explain the persistent encephalopathy observed outside periods of medication administration.

The optimal timing and utility of repeat MRI in heat stroke remain uncertain. This case underscores the limitations of conventional imaging and suggests that clinicians should maintain suspicion for significant neurologic injury even in the absence of MRI abnormalities. Repeat imaging with diffusion‐weighted, FLAIR, and susceptibility‐weighted sequences may be considered in patients with ongoing neurologic dysfunction once sedation and agitation permit.

## 4. Conclusion

This case illustrates that severe and persistent encephalopathy may occur in the setting of probable heat stroke despite normalization of body temperature and absence of acute abnormalities on conventional neuroimaging. While alternative etiologies cannot be fully excluded, the clinical course highlights the diagnostic uncertainty inherent in heat‐related neurologic injury and the limitations of MRI in detecting all forms of CNS damage. Clinicians should maintain a broad differential diagnosis and recognize that normal imaging does not preclude significant and potentially irreversible neurologic dysfunction.

## Funding

No funding was received for this manuscript.

## Conflicts of Interest

The authors declare no conflicts of interest.

## Data Availability

Data sharing not applicable to this article as no datasets were generated or analyzed during the current study.
